# 3α-Dimethyl­amino-20-(*N*-methyl­acetamido)­pregn-5-ene

**DOI:** 10.1107/S160053681103964X

**Published:** 2011-10-12

**Authors:** S. Yousuf, S. G. Musharraf, N. Iqbal, A. Adhikari, M. I. Choudhary

**Affiliations:** a H.E.J. Research Institute of Chemistry, International Center for Chemical and Biological Sciences, University of Karachi, Karachi 75270, Pakistan

## Abstract

The title compond, C_26_H_44_N_2_O, is an steroidal alkaloid isolated from the medicinally important plant *Sarcococca saligna*. The mol­ecule consists of four fused rings (*A*–*D*), having chair, half-chair, chair and envelope conformations, respectively. The dimethyl­amino group is axially oriented on ring *A*, whereas the (*N*-methyl­acetamido)­ethyl group is attached equatorially on ring *D*. The crystal structure is stabilized only by van der Waals forces.

## Related literature

For the biological activity of pregnane-type steroidal alkaloids isolated from plants belonging to the genus *Sarcococca*, see: Atta-ur-Rahman *et al.* (2000 ▶); Hassan *et al.* (2005 ▶); Kalauni *et al.* (2002 ▶); Naeem *et al.* (2005 ▶); Kiamuddin (1970 ▶); Kohli *et al.* (1964 ▶, 1967 ▶); Choudhary *et al.* (2004 ▶).
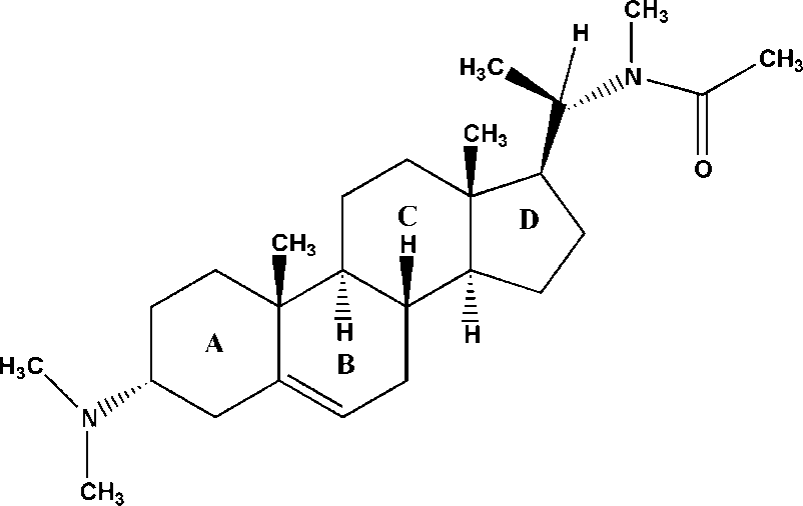


## Experimental

### 

#### Crystal data

C_26_H_44_N_2_O
*M* *_r_* = 400.63
Orthorhombic, 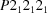

*a* = 6.1649 (5) Å
*b* = 11.9489 (9) Å
*c* = 31.982 (2) Å
*V* = 2355.9 (3) Å^3^
*Z* = 4
Mo *K*α radiation
μ = 0.07 mm^−1^
*T* = 273 K
0.32 × 0.13 × 0.13 mm

#### Data collection

Bruker SMART APEX CCD area-detector diffractometer
Absorption correction: multi-scan (*SADABS*; Bruker, 2009 ▶) *T*_min_ = 0.979, *T*_max_ = 0.991
14036 measured reflections
2559 independent reflections
2039 reflections with *I* > 2σ(*I*)
*R*_int_ = 0.044

#### Refinement

*R*[*F* ^2^ > 2σ(*F* ^2^)] = 0.044
*wR*(*F* ^2^) = 0.131
*S* = 1.12
2559 reflections
269 parameters
H-atom parameters constrained
Δρ_max_ = 0.17 e Å^−3^
Δρ_min_ = −0.17 e Å^−3^

### 

Data collection: *APEX2* (Bruker, 2009 ▶); cell refinement: *SAINT* (Bruker, 2009 ▶); data reduction: *SAINT*; program(s) used to solve structure: *SHELXS97* (Sheldrick, 2008 ▶); program(s) used to refine structure: *SHELXL97* (Sheldrick, 2008 ▶); molecular graphics: *SHELXTL* (Sheldrick, 2008 ▶); software used to prepare material for publication: *SHELXTL*, *PARST* (Nardelli, 1995 ▶) and *PLATON* (Spek, 2009 ▶).

## Supplementary Material

Crystal structure: contains datablock(s) global, I. DOI: 10.1107/S160053681103964X/rz2641sup1.cif

Structure factors: contains datablock(s) I. DOI: 10.1107/S160053681103964X/rz2641Isup2.hkl

Additional supplementary materials:  crystallographic information; 3D view; checkCIF report

## Figures and Tables

**Fig. 1.**
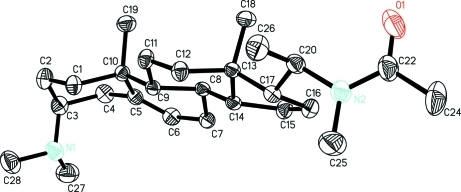
The molecular structure of the title compound, showing 30% probability displacement ellipsoids. H atoms are omitted for clarity.

**Fig. 2.**
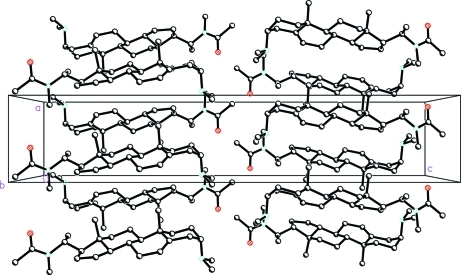
The crystal packing of the title compound viewed along the b axis.

## supplementary crystallographic information

### Comment

In the current study the title compound was isolated during the phytochemical investigation on the dichloromethane soluble part of a medicinally important plant *Sarcococca saligna* (D. Don.) Mull.-Arg. The leaves of this plant are locally used for the treatment of fever and rheumatism (Kiamuddin *et al.*, 1970). The pregnane-type steroidal alkaloids isolated from *Sarcococca saligna* and other plants belonging to genus *Sarcococca* are reported to have antispasmodic and cholinesterase inhibitory activities (Atta-ur-Rahman *et al.*, 2000; Hassan *et al.*, 2005; Kalauni *et al.*, 2002; Naeem *et al.*, 2005; Kiamuddin, 1970; Kohli *et al.*, 1964, 1967; Choudhary *et al.*, 2004).

The title compound (Fig. 1) is composed of four fused rings *A* (C1—C5/C10), *B* (C5—C10), *C* (C8—C9/C11—C14) and *D* (C13—C17). The trans fused rings *B* [Q= 0.472 (3) Å, θ = 130.3 (4)° and φ = 34.7 (4)°] and *C* [Q= 0.563 (3) Å, θ = 172.2 (3)° and φ = 87 (2)°] adopt half-chair and chair conformations, respectively, whereas ring *D* [Q= 0.446 (3)Å and φ = 9.1 (4)°] adopts an envelop conformation. Ring *A* exists in chair conformation [Q= 0.553 (3) Å, θ = 172.2 (3)° and φ = 249 (2)°] with an axially oriented dimethylamino group (N1/C25—C26) at C3. The (methylacetylamino)ethyl group is oriented equatorially at atom C17 of ring *D*. All bond lengths and angles are normal. In the crystal structure, the molecules are packed into parallel sheets along the *c* axis without any classical hydrogen bonds (Fig. 2).

### Experimental

Arial parts of *Sarcococca saligna* (D. Don.) Mull.-Arg. were collected at Swat, Pakistan, and identified by the taxonomist of the University of Karachi. A voucher specimen was deposited to the Herbarium of the University of Karachi (KU # 85854). Plants were dried under shade, weighted (50 kg), crushed and soacked in a methanol:water mixture (80:20 *v*/*v*) for one week, followed by filtration and evaporation under reduced pressure to yield 3 kg of syrupy crude extract. The methanolic extract was suspended in water and defatted with hexane. The water extract was basified with ammonium hydroxide to adjust the pH to 8–9 and then extracted with CH_2_Cl_2_ (3 × 5 *L*), to obtain the crude dichloromethane soluble part. This part was dried and subjected to silica gel vacuum liquid chromatography by using a gradient mixture of hexane/acetone/diethylamine (48:1:1 *v*/*v*/*v*) to afford several fractions (SSN10–17). The fraction SSN10 was further purified on alumina column by using an isocratic mobile phase of hexane/acetone/diethylamine (29:20:1 *v*/*v*/*v*) to afford the crystalline title compound. The compound was recrystalize by using a hexane/acetone solution (2:1 *v*/*v*) to afford colourless crystals suitable for single-crystal X-ray diffraction studies (5 mg).

### Refinement

H atoms were positioned geometrically with C–H = 0.93–0.98 Å and constrained to ride on their parent atoms with *U*_iso_(H) = 1.2*U*_eq_(C) or 1.5*U*_eq_(C) for methyl H atoms. A rotating group model was applied to the methyl groups. 1833 Friedel pairs were merged.

### Crystal data

**Table:**

C_26_H_44_N_2_O	*F*(000) = 888
*M**_r_* = 400.63	*D*_x_ = 1.130 Mg m^−^^3^
Orthorhombic, *P*2_1_2_1_2_1_	Mo *K*α radiation, λ = 0.71073 Å
Hall symbol: P 2ac 2ab	Cell parameters from 2160 reflections
*a* = 6.1649 (5) Å	θ = 2.6–20.2°
*b* = 11.9489 (9) Å	µ = 0.07 mm^−^^1^
*c* = 31.982 (2) Å	*T* = 273 K
*V* = 2355.9 (3) Å^3^	Block, colourless
*Z* = 4	0.32 × 0.13 × 0.13 mm

### Data collection

**Table:**

Bruker SMART APEX CCD area-detector diffractometer	2559 independent reflections
Radiation source: fine-focus sealed tube	2039 reflections with *I* > 2σ(*I*)
graphite	*R*_int_ = 0.044
ω scans	θ_max_ = 25.5°, θ_min_ = 1.3°
Absorption correction: multi-scan (*SADABS*; Bruker, 2009)	*h* = −7→7
*T*_min_ = 0.979, *T*_max_ = 0.991	*k* = −14→14
14036 measured reflections	*l* = −38→38

### Refinement

**Table:**

Refinement on *F*^2^	Primary atom site location: structure-invariant direct methods
Least-squares matrix: full	Secondary atom site location: difference Fourier map
*R*[*F*^2^ > 2σ(*F*^2^)] = 0.044	Hydrogen site location: inferred from neighbouring sites
*wR*(*F*^2^) = 0.131	H-atom parameters constrained
*S* = 1.12	*w* = 1/[σ^2^(*F*_o_^2^) + (0.0756*P*)^2^] where *P* = (*F*_o_^2^ + 2*F*_c_^2^)/3
2559 reflections	(Δ/σ)_max_ < 0.001
269 parameters	Δρ_max_ = 0.17 e Å^−^^3^
0 restraints	Δρ_min_ = −0.17 e Å^−^^3^

### Special details

**Table:**

Geometry. All e.s.d.'s (except the e.s.d. in the dihedral angle between two l.s. planes) are estimated using the full covariance matrix. The cell e.s.d.'s are taken into account individually in the estimation of e.s.d.'s in distances, angles and torsion angles; correlations between e.s.d.'s in cell parameters are only used when they are defined by crystal symmetry. An approximate (isotropic) treatment of cell e.s.d.'s is used for estimating e.s.d.'s involving l.s. planes.
Refinement. Refinement of *F*^2^ against ALL reflections. The weighted *R*-factor *wR* and goodness of fit *S* are based on *F*^2^, conventional *R*-factors *R* are based on *F*, with *F* set to zero for negative *F*^2^. The threshold expression of *F*^2^ > σ(*F*^2^) is used only for calculating *R*-factors(gt) *etc*. and is not relevant to the choice of reflections for refinement. *R*-factors based on *F*^2^ are statistically about twice as large as those based on *F*, and *R*- factors based on ALL data will be even larger.

### Fractional atomic coordinates and isotropic or equivalent isotropic displacement parameters (Å^2^)

**Table:**

	*x*	*y*	*z*	*U*_iso_*/*U*_eq_	
O1	0.6055 (6)	0.4028 (3)	0.45966 (9)	0.1050 (10)	
N1	0.9317 (5)	0.2814 (3)	0.07412 (7)	0.0618 (8)	
N2	0.8742 (4)	0.3158 (2)	0.42580 (7)	0.0547 (7)	
C1	0.6491 (5)	0.1990 (2)	0.14598 (8)	0.0489 (7)	
H1A	0.8039	0.1850	0.1474	0.059*	
H1B	0.5758	0.1364	0.1591	0.059*	
C2	0.5813 (6)	0.2050 (3)	0.10027 (9)	0.0596 (8)	
H2A	0.6146	0.1344	0.0867	0.072*	
H2B	0.4258	0.2164	0.0986	0.072*	
C3	0.6958 (5)	0.2989 (3)	0.07740 (9)	0.0547 (8)	
H3A	0.6362	0.3034	0.0490	0.066*	
C4	0.6407 (6)	0.4081 (2)	0.10031 (8)	0.0539 (8)	
H4A	0.7219	0.4688	0.0876	0.065*	
H4B	0.4876	0.4239	0.0965	0.065*	
C5	0.6901 (5)	0.4051 (2)	0.14659 (8)	0.0420 (6)	
C6	0.7992 (5)	0.4871 (2)	0.16484 (8)	0.0477 (7)	
H6A	0.8504	0.5444	0.1478	0.057*	
C7	0.8467 (5)	0.4951 (2)	0.21046 (8)	0.0432 (6)	
H7A	0.8164	0.5708	0.2198	0.052*	
H7B	0.9998	0.4809	0.2149	0.052*	
C8	0.7155 (4)	0.4136 (2)	0.23668 (8)	0.0357 (6)	
H8A	0.5681	0.4431	0.2398	0.043*	
C9	0.7029 (4)	0.29870 (19)	0.21472 (8)	0.0361 (6)	
H9A	0.8531	0.2748	0.2102	0.043*	
C10	0.5971 (4)	0.3065 (2)	0.17068 (8)	0.0388 (6)	
C11	0.5989 (5)	0.2097 (2)	0.24304 (8)	0.0453 (7)	
H11A	0.4447	0.2249	0.2450	0.054*	
H11B	0.6157	0.1371	0.2298	0.054*	
C12	0.6920 (5)	0.2033 (2)	0.28723 (8)	0.0439 (6)	
H12A	0.8399	0.1757	0.2859	0.053*	
H12B	0.6076	0.1506	0.3036	0.053*	
C13	0.6900 (4)	0.3176 (2)	0.30893 (8)	0.0350 (6)	
C14	0.8143 (4)	0.3982 (2)	0.28005 (8)	0.0366 (6)	
H14A	0.9565	0.3637	0.2755	0.044*	
C15	0.8566 (5)	0.5018 (2)	0.30672 (8)	0.0488 (7)	
H15A	0.7350	0.5532	0.3055	0.059*	
H15B	0.9866	0.5404	0.2975	0.059*	
C16	0.8852 (5)	0.4551 (2)	0.35099 (9)	0.0508 (7)	
H16A	0.7893	0.4935	0.3703	0.061*	
H16B	1.0335	0.4655	0.3603	0.061*	
C17	0.8288 (4)	0.3288 (2)	0.34926 (8)	0.0404 (6)	
H17A	0.9644	0.2879	0.3446	0.048*	
C18	0.4553 (4)	0.3558 (3)	0.31689 (9)	0.0477 (7)	
H18A	0.3803	0.2998	0.3328	0.072*	
H18B	0.3827	0.3667	0.2906	0.072*	
H18C	0.4563	0.4250	0.3322	0.072*	
C19	0.3490 (5)	0.3228 (3)	0.17399 (10)	0.0547 (8)	
H19A	0.2899	0.3356	0.1466	0.082*	
H19B	0.3185	0.3860	0.1915	0.082*	
H19C	0.2845	0.2569	0.1858	0.082*	
C20	0.7320 (5)	0.2866 (2)	0.39047 (8)	0.0464 (7)	
H20A	0.5947	0.3263	0.3947	0.056*	
C22	0.7953 (7)	0.3729 (3)	0.45846 (10)	0.0647 (9)	
C23	0.9450 (8)	0.3975 (4)	0.49421 (10)	0.0925 (14)	
H23A	0.8721	0.4443	0.5142	0.139*	
H23B	1.0717	0.4354	0.4840	0.139*	
H23C	0.9871	0.3287	0.5074	0.139*	
C24	1.0933 (6)	0.2691 (4)	0.42659 (12)	0.0872 (13)	
H24A	1.1216	0.2376	0.4537	0.131*	
H24B	1.1965	0.3273	0.4209	0.131*	
H24C	1.1056	0.2118	0.4057	0.131*	
C21	0.6810 (7)	0.1615 (3)	0.39121 (9)	0.0644 (9)	
H21A	0.6454	0.1392	0.4192	0.097*	
H21B	0.8053	0.1203	0.3818	0.097*	
H21C	0.5603	0.1465	0.3731	0.097*	
C25	1.0361 (7)	0.3708 (4)	0.05107 (13)	0.0892 (13)	
H25A	1.0205	0.4398	0.0661	0.134*	
H25B	0.9695	0.3780	0.0241	0.134*	
H25C	1.1873	0.3540	0.0477	0.134*	
C26	0.9805 (7)	0.1756 (4)	0.05331 (11)	0.0854 (12)	
H26A	0.9357	0.1145	0.0708	0.128*	
H26B	1.1337	0.1706	0.0483	0.128*	
H26C	0.9042	0.1721	0.0272	0.128*	

### Atomic displacement parameters (Å^2^)

**Table:**

	*U*^11^	*U*^22^	*U*^33^	*U*^12^	*U*^13^	*U*^23^
O1	0.094 (2)	0.146 (3)	0.0749 (18)	0.024 (2)	0.0099 (17)	−0.0450 (18)
N1	0.0633 (17)	0.0799 (19)	0.0422 (14)	0.0128 (16)	−0.0039 (13)	−0.0085 (14)
N2	0.0550 (15)	0.0679 (16)	0.0412 (13)	0.0036 (14)	−0.0018 (12)	−0.0029 (12)
C1	0.0605 (19)	0.0406 (14)	0.0457 (15)	0.0008 (15)	−0.0008 (15)	−0.0013 (13)
C2	0.073 (2)	0.0580 (18)	0.0473 (16)	−0.0024 (18)	−0.0072 (16)	−0.0085 (16)
C3	0.0622 (19)	0.065 (2)	0.0364 (15)	0.0071 (19)	−0.0114 (15)	−0.0019 (14)
C4	0.0619 (19)	0.0528 (17)	0.0468 (16)	0.0073 (17)	−0.0004 (16)	0.0098 (14)
C5	0.0452 (15)	0.0373 (14)	0.0434 (14)	0.0080 (14)	0.0033 (14)	0.0026 (12)
C6	0.0546 (17)	0.0397 (15)	0.0489 (16)	−0.0021 (15)	0.0076 (15)	0.0111 (13)
C7	0.0451 (15)	0.0327 (13)	0.0517 (16)	−0.0037 (13)	0.0068 (14)	−0.0003 (12)
C8	0.0317 (13)	0.0314 (12)	0.0441 (14)	0.0028 (12)	0.0030 (12)	−0.0013 (11)
C9	0.0355 (13)	0.0298 (12)	0.0429 (14)	0.0001 (12)	0.0043 (12)	−0.0018 (11)
C10	0.0397 (14)	0.0355 (14)	0.0413 (14)	0.0023 (12)	−0.0025 (12)	−0.0011 (12)
C11	0.0573 (18)	0.0343 (13)	0.0442 (15)	−0.0082 (14)	0.0008 (13)	−0.0024 (12)
C12	0.0540 (16)	0.0342 (13)	0.0434 (14)	−0.0033 (14)	0.0023 (14)	0.0004 (12)
C13	0.0314 (13)	0.0339 (13)	0.0398 (13)	0.0014 (12)	0.0018 (11)	−0.0013 (11)
C14	0.0303 (12)	0.0330 (13)	0.0464 (14)	0.0023 (12)	0.0033 (12)	−0.0038 (11)
C15	0.0549 (18)	0.0397 (14)	0.0518 (16)	−0.0070 (15)	−0.0001 (14)	−0.0058 (13)
C16	0.0500 (17)	0.0525 (17)	0.0499 (16)	−0.0076 (15)	−0.0019 (15)	−0.0097 (14)
C17	0.0375 (14)	0.0424 (14)	0.0413 (14)	0.0027 (13)	0.0036 (13)	−0.0032 (12)
C18	0.0367 (15)	0.0564 (17)	0.0500 (16)	0.0015 (13)	0.0052 (13)	0.0007 (13)
C19	0.0434 (16)	0.0641 (19)	0.0567 (17)	0.0002 (16)	−0.0026 (15)	0.0004 (15)
C20	0.0455 (16)	0.0562 (17)	0.0374 (14)	0.0056 (14)	0.0034 (12)	−0.0022 (13)
C22	0.079 (3)	0.072 (2)	0.0423 (18)	−0.005 (2)	0.0053 (19)	−0.0065 (16)
C23	0.128 (4)	0.101 (3)	0.0485 (19)	−0.024 (3)	−0.009 (2)	−0.015 (2)
C24	0.062 (2)	0.135 (4)	0.064 (2)	0.024 (3)	−0.0118 (19)	−0.007 (2)
C21	0.082 (2)	0.065 (2)	0.0463 (17)	−0.016 (2)	0.0052 (18)	0.0053 (15)
C25	0.072 (3)	0.104 (3)	0.092 (3)	−0.005 (3)	0.010 (2)	0.001 (2)
C26	0.089 (3)	0.101 (3)	0.066 (2)	0.024 (3)	−0.005 (2)	−0.021 (2)

### Geometric parameters (Å, °)

**Table:**

O1—C22	1.224 (5)	C12—H12B	0.9700
N1—C25	1.449 (5)	C13—C18	1.539 (4)
N1—C26	1.460 (4)	C13—C14	1.539 (3)
N1—C3	1.473 (4)	C13—C17	1.554 (3)
N2—C22	1.339 (4)	C14—C15	1.526 (3)
N2—C24	1.461 (4)	C14—H14A	0.9800
N2—C20	1.472 (4)	C15—C16	1.532 (4)
C1—C2	1.522 (4)	C15—H15A	0.9700
C1—C10	1.542 (4)	C15—H15B	0.9700
C1—H1A	0.9700	C16—C17	1.549 (4)
C1—H1B	0.9700	C16—H16A	0.9700
C2—C3	1.514 (5)	C16—H16B	0.9700
C2—H2A	0.9700	C17—C20	1.532 (4)
C2—H2B	0.9700	C17—H17A	0.9800
C3—C4	1.534 (4)	C18—H18A	0.9600
C3—H3A	0.9800	C18—H18B	0.9600
C4—C5	1.512 (4)	C18—H18C	0.9600
C4—H4A	0.9700	C19—H19A	0.9600
C4—H4B	0.9700	C19—H19B	0.9600
C5—C6	1.324 (4)	C19—H19C	0.9600
C5—C10	1.521 (4)	C20—C21	1.527 (4)
C6—C7	1.491 (4)	C20—H20A	0.9800
C6—H6A	0.9300	C22—C23	1.498 (5)
C7—C8	1.519 (3)	C23—H23A	0.9600
C7—H7A	0.9700	C23—H23B	0.9600
C7—H7B	0.9700	C23—H23C	0.9600
C8—C14	1.526 (3)	C24—H24A	0.9600
C8—C9	1.544 (3)	C24—H24B	0.9600
C8—H8A	0.9800	C24—H24C	0.9600
C9—C11	1.537 (4)	C21—H21A	0.9600
C9—C10	1.555 (4)	C21—H21B	0.9600
C9—H9A	0.9800	C21—H21C	0.9600
C10—C19	1.545 (4)	C25—H25A	0.9600
C11—C12	1.528 (4)	C25—H25B	0.9600
C11—H11A	0.9700	C25—H25C	0.9600
C11—H11B	0.9700	C26—H26A	0.9600
C12—C13	1.533 (3)	C26—H26B	0.9600
C12—H12A	0.9700	C26—H26C	0.9600
			
C25—N1—C26	108.4 (3)	C14—C13—C17	99.8 (2)
C25—N1—C3	111.7 (3)	C15—C14—C8	118.6 (2)
C26—N1—C3	111.0 (3)	C15—C14—C13	104.9 (2)
C22—N2—C24	121.1 (3)	C8—C14—C13	115.0 (2)
C22—N2—C20	120.2 (3)	C15—C14—H14A	105.8
C24—N2—C20	118.2 (3)	C8—C14—H14A	105.8
C2—C1—C10	113.3 (2)	C13—C14—H14A	105.8
C2—C1—H1A	108.9	C14—C15—C16	103.9 (2)
C10—C1—H1A	108.9	C14—C15—H15A	111.0
C2—C1—H1B	108.9	C16—C15—H15A	111.0
C10—C1—H1B	108.9	C14—C15—H15B	111.0
H1A—C1—H1B	107.7	C16—C15—H15B	111.0
C3—C2—C1	111.7 (3)	H15A—C15—H15B	109.0
C3—C2—H2A	109.3	C15—C16—C17	107.2 (2)
C1—C2—H2A	109.3	C15—C16—H16A	110.3
C3—C2—H2B	109.3	C17—C16—H16A	110.3
C1—C2—H2B	109.3	C15—C16—H16B	110.3
H2A—C2—H2B	107.9	C17—C16—H16B	110.3
N1—C3—C2	112.9 (3)	H16A—C16—H16B	108.5
N1—C3—C4	111.9 (3)	C20—C17—C16	112.2 (2)
C2—C3—C4	107.2 (2)	C20—C17—C13	118.1 (2)
N1—C3—H3A	108.2	C16—C17—C13	103.7 (2)
C2—C3—H3A	108.2	C20—C17—H17A	107.4
C4—C3—H3A	108.2	C16—C17—H17A	107.4
C5—C4—C3	113.8 (2)	C13—C17—H17A	107.4
C5—C4—H4A	108.8	C13—C18—H18A	109.5
C3—C4—H4A	108.8	C13—C18—H18B	109.5
C5—C4—H4B	108.8	H18A—C18—H18B	109.5
C3—C4—H4B	108.8	C13—C18—H18C	109.5
H4A—C4—H4B	107.7	H18A—C18—H18C	109.5
C6—C5—C4	121.1 (2)	H18B—C18—H18C	109.5
C6—C5—C10	122.8 (2)	C10—C19—H19A	109.5
C4—C5—C10	116.0 (2)	C10—C19—H19B	109.5
C5—C6—C7	125.4 (2)	H19A—C19—H19B	109.5
C5—C6—H6A	117.3	C10—C19—H19C	109.5
C7—C6—H6A	117.3	H19A—C19—H19C	109.5
C6—C7—C8	113.2 (2)	H19B—C19—H19C	109.5
C6—C7—H7A	108.9	N2—C20—C21	110.0 (3)
C8—C7—H7A	108.9	N2—C20—C17	110.5 (2)
C6—C7—H7B	108.9	C21—C20—C17	114.6 (2)
C8—C7—H7B	108.9	N2—C20—H20A	107.1
H7A—C7—H7B	107.7	C21—C20—H20A	107.1
C7—C8—C14	111.5 (2)	C17—C20—H20A	107.1
C7—C8—C9	110.2 (2)	O1—C22—N2	121.4 (3)
C14—C8—C9	109.03 (19)	O1—C22—C23	120.5 (4)
C7—C8—H8A	108.7	N2—C22—C23	118.2 (4)
C14—C8—H8A	108.7	C22—C23—H23A	109.5
C9—C8—H8A	108.7	C22—C23—H23B	109.5
C11—C9—C8	111.6 (2)	H23A—C23—H23B	109.5
C11—C9—C10	113.6 (2)	C22—C23—H23C	109.5
C8—C9—C10	112.3 (2)	H23A—C23—H23C	109.5
C11—C9—H9A	106.2	H23B—C23—H23C	109.5
C8—C9—H9A	106.2	N2—C24—H24A	109.5
C10—C9—H9A	106.2	N2—C24—H24B	109.5
C5—C10—C1	107.9 (2)	H24A—C24—H24B	109.5
C5—C10—C19	108.1 (2)	N2—C24—H24C	109.5
C1—C10—C19	110.3 (2)	H24A—C24—H24C	109.5
C5—C10—C9	110.3 (2)	H24B—C24—H24C	109.5
C1—C10—C9	109.1 (2)	C20—C21—H21A	109.5
C19—C10—C9	111.2 (2)	C20—C21—H21B	109.5
C12—C11—C9	115.0 (2)	H21A—C21—H21B	109.5
C12—C11—H11A	108.5	C20—C21—H21C	109.5
C9—C11—H11A	108.5	H21A—C21—H21C	109.5
C12—C11—H11B	108.5	H21B—C21—H21C	109.5
C9—C11—H11B	108.5	N1—C25—H25A	109.5
H11A—C11—H11B	107.5	N1—C25—H25B	109.5
C11—C12—C13	111.8 (2)	H25A—C25—H25B	109.5
C11—C12—H12A	109.3	N1—C25—H25C	109.5
C13—C12—H12A	109.3	H25A—C25—H25C	109.5
C11—C12—H12B	109.3	H25B—C25—H25C	109.5
C13—C12—H12B	109.3	N1—C26—H26A	109.5
H12A—C12—H12B	107.9	N1—C26—H26B	109.5
C12—C13—C18	110.3 (2)	H26A—C26—H26B	109.5
C12—C13—C14	106.4 (2)	N1—C26—H26C	109.5
C18—C13—C14	112.4 (2)	H26A—C26—H26C	109.5
C12—C13—C17	116.6 (2)	H26B—C26—H26C	109.5
C18—C13—C17	110.8 (2)		
			
C10—C1—C2—C3	60.5 (4)	C9—C11—C12—C13	53.2 (3)
C25—N1—C3—C2	178.3 (3)	C11—C12—C13—C18	66.7 (3)
C26—N1—C3—C2	57.2 (3)	C11—C12—C13—C14	−55.5 (3)
C25—N1—C3—C4	−60.5 (3)	C11—C12—C13—C17	−165.8 (2)
C26—N1—C3—C4	178.4 (2)	C7—C8—C14—C15	54.2 (3)
C1—C2—C3—N1	65.6 (3)	C9—C8—C14—C15	176.1 (2)
C1—C2—C3—C4	−58.2 (3)	C7—C8—C14—C13	179.4 (2)
N1—C3—C4—C5	−70.5 (4)	C9—C8—C14—C13	−58.6 (3)
C2—C3—C4—C5	53.8 (4)	C12—C13—C14—C15	−166.8 (2)
C3—C4—C5—C6	131.5 (3)	C18—C13—C14—C15	72.4 (3)
C3—C4—C5—C10	−51.7 (4)	C17—C13—C14—C15	−45.1 (2)
C4—C5—C6—C7	176.7 (3)	C12—C13—C14—C8	61.1 (3)
C10—C5—C6—C7	0.1 (5)	C18—C13—C14—C8	−59.7 (3)
C5—C6—C7—C8	−13.1 (4)	C17—C13—C14—C8	−177.20 (19)
C6—C7—C8—C14	162.5 (2)	C8—C14—C15—C16	162.9 (2)
C6—C7—C8—C9	41.2 (3)	C13—C14—C15—C16	32.9 (3)
C7—C8—C9—C11	172.1 (2)	C14—C15—C16—C17	−7.2 (3)
C14—C8—C9—C11	49.4 (3)	C15—C16—C17—C20	−149.0 (3)
C7—C8—C9—C10	−59.1 (3)	C15—C16—C17—C13	−20.5 (3)
C14—C8—C9—C10	178.2 (2)	C12—C13—C17—C20	−81.8 (3)
C6—C5—C10—C1	−135.5 (3)	C18—C13—C17—C20	45.5 (3)
C4—C5—C10—C1	47.7 (3)	C14—C13—C17—C20	164.2 (2)
C6—C5—C10—C19	105.3 (3)	C12—C13—C17—C16	153.4 (2)
C4—C5—C10—C19	−71.5 (3)	C18—C13—C17—C16	−79.3 (3)
C6—C5—C10—C9	−16.4 (4)	C14—C13—C17—C16	39.4 (2)
C4—C5—C10—C9	166.8 (2)	C22—N2—C20—C21	−107.1 (3)
C2—C1—C10—C5	−51.7 (3)	C24—N2—C20—C21	65.3 (4)
C2—C1—C10—C19	66.1 (3)	C22—N2—C20—C17	125.3 (3)
C2—C1—C10—C9	−171.6 (2)	C24—N2—C20—C17	−62.2 (4)
C11—C9—C10—C5	173.2 (2)	C16—C17—C20—N2	−52.8 (3)
C8—C9—C10—C5	45.4 (3)	C13—C17—C20—N2	−173.4 (2)
C11—C9—C10—C1	−68.4 (3)	C16—C17—C20—C21	−177.8 (3)
C8—C9—C10—C1	163.7 (2)	C13—C17—C20—C21	61.6 (3)
C11—C9—C10—C19	53.4 (3)	C24—N2—C22—O1	−172.9 (4)
C8—C9—C10—C19	−74.5 (3)	C20—N2—C22—O1	−0.6 (5)
C8—C9—C11—C12	−49.2 (3)	C24—N2—C22—C23	6.0 (5)
C10—C9—C11—C12	−177.4 (2)	C20—N2—C22—C23	178.2 (3)
